# Split green fluorescent protein as a modular binding partner for protein crystallization

**DOI:** 10.1107/S0907444913024608

**Published:** 2013-11-19

**Authors:** Hau B. Nguyen, Li-Wei Hung, Todd O. Yeates, Thomas C. Terwilliger, Geoffrey S. Waldo

**Affiliations:** a Bioscience Division, Los Alamos National Laboratory, MS M888, Los Alamos, NM 87545, USA; b Physics Division, Los Alamos National Laboratory, MS D454, Los Alamos, NM 87545, USA; c Department of Chemistry and Biochemistry, University of California, PO Box 951569, Los Angeles, CA 90095, USA

**Keywords:** protein crystallization, synthetic symmetrization, protein tagging, split GFP, split protein, green fluorescent protein, protein expression, protein-fragment complementation, crystallization reagents

## Abstract

A modular strategy for protein crystallization using split green fluorescent protein (GFP) as a crystallization partner is demonstrated. Insertion of a hairpin containing GFP β-­strands 10 and 11 into a surface loop of a target protein provides two chain crossings between the target and the reconstituted GFP compared with the single connection afforded by terminal GFP fusions. This strategy was tested by inserting this hairpin into a loop of another fluorescent protein, sfCherry. The crystal structure of the sfCherry-GFP(10–11) hairpin in complex with GFP(1–9) was determined at a resolution of 2.6 Å. Analysis of the complex shows that the reconstituted GFP is attached to the target protein (sfCherry) in a structurally ordered way. This work opens the way to rapidly creating crystallization variants by reconstituting a target protein bearing the GFP(10–11) hairpin with a variety of GFP(1–9) mutants engineered for favorable crystallization.

## Abstract 2

A strategy using a new split green fluorescent protein (GFP) as a modular binding partner to form stable protein complexes with a target protein is presented. The modular split GFP may open the way to rapidly creating crystallization variants.

## 1. Introduction   

Structural characterization of proteins, protein complexes and small molecules is essential to understand cellular functions from enzymology to macromolecular machines. Knowledge of protein structures has led to the redesign of protein function and folding using rational and semi-rational approaches, and has promoted the discovery of new and improved small-molecule drugs (Lu *et al.*, 2009 ▶; Yeung *et al.*, 2009 ▶; Lin *et al.*, 2010 ▶). Yet obtaining well ordered crystals, a prerequisite of macromolecular crystallography, remains a major obstacle; as many as 70% of purified proteins fail to crystallize (Terwilliger *et al.*, 2009 ▶).

A number of current approaches to improve protein crystallization involve constructing variant forms of the target protein molecule. Examples include engineering proteins with enhanced solubility by site-directed mutagenesis (Nasreen *et al.*, 2006 ▶; Eichinger *et al.*, 2007 ▶) or directed evolution (Farinas *et al.*, 2001 ▶; Pédelacq *et al.*, 2002 ▶; Waldo, 2003 ▶; Cabantous, Pedelacq *et al.*, 2005 ▶) and the removal of disordered regions, often at the N- or C-terminus (Thornton & Sibanda, 1983 ▶), by proteolysis (Dong *et al.*, 2007 ▶) or targeted deletion (Pantazatos *et al.*, 2004 ▶) based on disorder prediction. Proteins may also contain internally disordered regions such as loops or subdomains, which can sometimes be removed, shortened or replaced by a short linker to reduce conformational heterogeneity, thereby increasing crystallization propensity (Kwong *et al.*, 1998 ▶, 1999 ▶; Derewenda, 2010 ▶). Other methods such as surface-entropy reduction (Longenecker *et al.*, 2001 ▶; Derewenda, 2004 ▶; Cooper *et al.*, 2007 ▶) and lysine methylation (Rypniewski *et al.*, 1993 ▶; Walter *et al.*, 2006 ▶; Kim *et al.*, 2008 ▶) drive crystallization by changing the surface properties of proteins and promoting lattice contacts. The surface-entropy reduction method has been successfully applied not only to individual proteins but also to protein–protein complexes and membrane proteins (Berman *et al.*, 2007 ▶; Levinson *et al.*, 2008 ▶; Yanez *et al.*, 2008 ▶; Pornillos *et al.*, 2009 ▶; Yip *et al.*, 2005 ▶).

Other methods for modifying and potentially improving the crystallization properties of a protein involve connecting it to another protein intended to act as a carrier. Highly soluble proteins have been used as fusion partners to the N-terminus or C-terminus of proteins to enhance their folding and solubility and to mediate crystal contacts (Wiltzius *et al.*, 2009 ▶; Kuge *et al.*, 1997 ▶; Center *et al.*, 1998 ▶; Monné *et al.*, 2008 ▶; Ullah *et al.*, 2008 ▶; Smyth *et al.*, 2003 ▶; Moon *et al.*, 2010 ▶). Carrier proteins have been inserted into loops of transmembrane proteins (Engel *et al.*, 2002 ▶) and the insertion of T4 lysozyme into a loop of the β_2_-adrenergic receptor is an example of a successful application of this strategy (Rosenbaum *et al.*, 2007 ▶; Cherezov *et al.*, 2007 ▶). Noncovalent crystallization chaperones such as Fab and Fv fragments of antibodies (Kovari *et al.*, 1995 ▶; Lange & Hunte, 2002 ▶; Lee *et al.*, 2005 ▶; Ostermeier *et al.*, 1995 ▶; Monroe *et al.*, 2011 ▶) and designed ankyrin-repeat protein (DARPin; Monroe *et al.*, 2011 ▶) have alternatively been used to produce complexes with target molecules. These complexes often show improved solubility and crystallizability in comparison to the isolated targets (Derewenda, 2010 ▶).

Synthetic symmetrization of proteins offers a further approach to expand crystallization opportunities. Variant forms of a target protein molecule are constructed, with each designed to produce a structurally distinct oligomer. Disulfide-based synthetic dimerization (Banatao *et al.*, 2006 ▶; Forse *et al.*, 2011 ▶) and designed metal-mediated oligomerization have both been demonstrated (Laganowsky *et al.*, 2011 ▶). Other examples using different motifs such as leucine zippers to drive the self-­association of a target protein have also been shown to promote protein symmetrization and crystallization (Yamada *et al.*, 2007 ▶).

With current strategies for expanding the crystallization opportunities for a target protein, the effort required to produce many structural variants is a major challenge. A modular approach could offer important advantages. In particular, an ideal strategy might factor the problem of repeatedly re-engineering a protein of interest into two separate problems: (i) connecting the target protein to a carrier protein and (ii) creating variant forms of the carrier protein. In order to fully separate the two problems, the connection between the target protein and the carrier protein should occur by noncovalent molecular recognition rather than by genetic covalent attachment, so that repeated genetic modification and purification of the protein of interest can be avoided. Additionally, the target protein and the carrier protein should ideally be attached in a way that minimizes the flexibility between them, as too much flexibility would reduce the chances of forming well ordered crystals of the complex. Finally, the structural feature that drives the noncovalent association between the target protein and the carrier protein should ideally be transferable from one target system to another. In this way, one set of variational forms of the carrier protein can be utilized, without continual re-engineering, for a range of target proteins.

In this work, we demonstrate a system that meets the design requirements above and is based on green fluorescent protein (GFP). GFP has been employed before in crystallization experiments based on protein fusions (Suzuki *et al.*, 2010 ▶). Also, previous studies on GFP have shown that it can form the basis for a complementation system: fragments composed of either β-strand 11 or a hairpin comprised of β-strands 10 and 11 can reassemble with truncated forms of GFP lacking these segments (Cabantous, Terwilliger *et al.*, 2005 ▶; Cabantous *et al.*, 2013 ▶). We show here that β-­strands 10 and 11 of GFP can be inserted as a hairpin into a protruding loop of a target protein, which when complemented by GFP(1–9) gives rise to a well ordered complex with two polypeptide-chain crossings between the two components which is amenable to crystal structure analysis. Prospects for developing the system for general applications are discussed.

## 2. Methods   

### 2.1. Engineering superfolder Cherry (sfCherry)   

The monomeric fluorescent protein Cherry (Shaner *et al.*, 2004 ▶) was cloned as a C-terminal fusion to ferritin in a modified pET expression plasmid as described by Pédelacq *et al.* (2006 ▶). The N-terminal and C-terminal GFP sequence extensions (residues MVSKG and MDELYK, respectively; Supplementary Fig. S2*b* 1) that were added to improve mCherry protein solubility in an earlier study (Shaner *et al.*, 2004 ▶) were omitted here to increase the stringency of selection for better solubility and stability. The DNA encoding mCherry was amplified by PCR using vector flanking primers and was subjected to DNA fragmentation and shuffling using published protocols (Stemmer, 1994 ▶). The cDNA library plasmid pool was transformed into *Escherichia coli* BL21 (DE3) Gold (Novagen) competent cells for protein expression. The library was plated on nitrocellulose membranes using two sequential 400-fold dilutions of a 1.0 OD_600 nm_ cell stock frozen in 20% glycerol/Luria–Bertani (LB), yielding ∼3 × 10^3^ colonies per plate. Cells were grown overnight at 305 K and proteins were expressed by transferring the membrane to an LB–agar plate containing 35 µg kanamycin per millilitre of medium and 1 m*M* isopropyl β-d-1-thio­galactopyranoside (IPTG) for 3 h at 310 K. Clones displaying the brightest fluorescence (550 nm excitation/610 nm emission) were selected, grown overnight and frozen in 20% glycerol/LB freezer stocks at 193 K. These brightest clones were selected as templates for the next round of evolution. After three rounds of directed evolution, the sequences of the constructs were confirmed by DNA sequencing and the brightest clone coding sfCherry was chosen.

### 2.2. Insertion of the GFP strands 10–11 hairpin and selection of a clone with a permissive loop   

GFP strands 10–11 (DLPD**DHYLSTQTILS**KDLNEKRD**HMVLLEYVTAA**GIT*DAS*, with residues in strand 10 and strand 11 shown in bold and those in the three-residue linker DAS italicized) were inserted into permissive loops of sfCherry by PCR. Primer sequences are included in Supplementary Table S1. Fragments were cloned into pTET ColE1 vector and transformed into *E. coli* BL21 (DE3) competent cells containing pET GFP strands 1–9 for *in vivo* testing. *In vivo* protein expression and solubility screenings were performed as described previously (Cabantous & Waldo, 2006 ▶). 1 OD_600 nm_ frozen cell stocks in 20% glycerol/LB were thawed and diluted 400-fold (twice) in LB and plated onto a nitrocellulose membrane with selective LB–agar containing 35 µg ml^−1^ kanamycin (Kan) and 75 µg ml^−1^ spectinomycin (Spec). After overnight growth at 305 K, the membrane was transferred to a pre-warmed plate containing 0.3 µg ml^−1^ anhydrotetracycline (AnTet), 1 m*M* IPTG for 4 h at 303 K for protein expression screening. For protein solubility testing, the membrane was transferred to a pre-warmed plate containing 0.3 µg ml^−1^ AnTet for 2 h, rested back to its original LB–Kan–Spec plate for 1 h to allow the AnTet to diffuse out and followed by induction on an LB–Kan–Spec plate with 1 m*M* IPTG at 303 K for 1 h. The induced plates were illuminated using an Illumatool Lighting System (LightTools Research) equipped with 488/520 nm (for GFP) and 550/610 nm (for sfCherry) excitation/emission filters.

### 2.3. Expression and refolding of GFP(1–9) and GFP(1–10) fragments   

GFP(1–9) and GFP(1–10) proteins were expressed and prepared as described previously (Cabantous, Terwilliger *et al.*, 2005 ▶; Cabantous & Waldo, 2006 ▶). Briefly, 1 l cultures of *E. coli* BL21(DE3) cells expressing GFP(1–9) or GFP(1–10) constructs were grown until an OD_600 nm_ of 0.5–0.7 was reached, protein expression was induced with 1 m*M* IPTG and the cells were harvested after 5 h of induction at 310 K. The harvested cells were resuspended in 50 m*M* Tris pH 7.4, 0.1 *M* NaCl, 10% glycerol (TNG buffer) and lysed by sonication on ice. Inclusion bodies containing GFP(1–10) and GFP(1–9) were recovered by centrifugation at 20 000*g*. Inclusion bodies were washed and prepared in individual Eppendorf tubes (∼75 mg inclusion bodies per tube) as described previously (Cabantous & Waldo, 2006 ▶). Prepared inclusion bodies can be stored at 193 K for at least several months. 75 mg of the washed inclusion bodies prepared in a 1.5 ml Eppendorf tube was unfolded with 1 ml 9 *M* urea in TNG buffer and refolded by adding 25 volumes of TNG buffer. The soluble solutions were filtered through a 0.2 µm syringe filter and the protein was quantified using the Bio-Rad Protein Assay reagent (Bio-Rad). This refolded protein solution is ready for protein complementation and can also be stored for up to a week at 253 K for later use.

### 2.4. Expression and purification of sfCherry and sfCherry-GFP(10–11)–GFP(1–9) complex   

sfCherry with GFP strands 10–11 inserted at position Asp169/Gly170 was subcloned into pET with a noncleavable C-terminal His_6_ tag. The C-terminal in-frame *Bam*HI site introduced a GS amino-acid motif between sfCherry and the His_6_ tag. Proteins were expressed in *E. coli* BL21(DE3) cells under the control of the IPTG-inducible T7 promoter. A 1 l culture of *E. coli* BL21(DE3) cells expressing sfCherry or sfCherry with GFP strand 10–11 inserted was grown to an OD_600 nm_ of ∼0.5–0.7 and induced with 1 m*M* IPTG for 7 h at 303 K. The harvested cells were suspended in TNG buffer and lysed by sonication on ice for 10 min at 70% duty cycle. The mixture was then centrifuged at 15 000*g* for 30 min at 283 K to remove cell debris. For sfCherry and sfCherry-GFP(10–11) the supernatant was incubated with pre-equilibrated Talon metal-affinity resin (Clontech) and the mixture was incubated at room temperature with gentle shaking for 1 h to allow the protein to bind to the resin. The protein bound to the resin was separated from unbound protein by centrifugation at 3000*g* for 5 min and the resin was washed two times with column buffer before it was packed into a gravity-flow column. The column was then washed with 50 ml column buffer (50 m*M* sodium phosphate buffer pH 7, 300 m*M* NaCl, 10% glycerol) followed by 50 ml binding buffer (50 m*M* sodium phosphate buffer pH 7, 300 m*M* NaCl, 10% glycerol, 5 m*M* imidazole) and 20 ml washing buffer (50 m*M* sodium phosphate buffer pH 7, 300 m*M* NaCl, 10% glycerol, 20 m*M* imidazole) to remove unbound and nonspecifically bound proteins, respectively. The purified proteins were completely eluted with 250 m*M* imidazole in TNG buffer with a good yield of about 40 mg per litre of cell culture. The protein solutions were concentrated and exchanged to final buffer [20 m*M* Tris–HCl pH 8, 150 m*M* NaCl, 1 m*M* dithiothreitol (DTT)] using an Amicon Ultra-15 centrifugal filter device (10 kDa cutoff; Millipore).

To create the sfCherry-GFP(10–11)–GFP(1–9) protein complex, purified sfCherry-GFP(10–11) was complemented overnight in the cold room with an excess amount of refolded GFP(1–9) such that the amount of GFP(1–9) was not limiting. The protein mixture was applied onto pre-equilibrated Talon metal-affinity resin and the protein complex was subsequently purified using the same purification protocol used for sfCherry and sfCherry-GFP(10–11) as indicated above. For each purification step, the protein elution samples were resolved on a 4–­20% gradient Criterion SDS–PAGE gel (Bio-Rad, Hercules, California, USA) and stained using Gel Code Blue stain reagent (Pierce, Rockford, Illinois, USA).

### 2.5. *In vitro* complementation assays of sfCherry-GFP(10–11) (Asp169/Gly170) with GFP(1–9) or GFP(1–10)   

*In vitro* complementation assays were performed as described previously (Cabantous & Waldo, 2006 ▶). A 96-well microplate (Nunc-Immuno plate, Nunc) was first blocked with a solution of 0.5% bovine serum albumin (BSA) in TNG for 10 min. Purified sfCherry-GFP(10–11) hairpin was subjected to twofold serial dilutions in the same buffer so that the dilutions spanned the range 1–200 pmol per 20 µl aliquot. Protein aliquots were added to 96-well plates and complementation was performed using a large excess of GFP(1–9) or GFP(1–10) (∼1 mg ml^−1^, 800 pmol) added in a 180 µl aliquot. Fluorescence kinetics (488 nm excitation/520 nm emission) were monitored with a DTX Microplate Fluorescence Reader (Beckman Coulter) at 3 min intervals for 15 h. The background fluorescence of a blank sample [20 µl 0.5% BSA in TNG buffer, 180 µl 1 mg ml^−1^ GFP(1–9) or GFP(1–10) in TNG buffer] was subtracted from the final fluorescence values.

### 2.6. Crystallization   

SfCherry (at a concentration of ∼25 mg ml^−1^) and the sfCherry-GFP(10–11)–GFP(1–9) complex (at a concentration of ∼22 mg ml^−1^) were both crystallized using the sitting-drop vapor-diffusion method by mixing 0.15 µl protein stock with 0.15 µl reservoir solution and equilibrating the drop against 30 µl reservoir solution at 298 K. A set of 384 crystallization reagents consisting of Crystal Screen, Crystal Screen 2 (Hampton Research), PACT suite (Qiagen) and JCSG Core Suites I and II (Qiagen) was used to screen for the propensity of crystallization. Subsequent optimization fine-tuning of pH, salt, precipitants and additives were employed as needed until diffraction-quality crystals were obtained.

For sfCherry crystallization, six conditions from the initial screening, including four closely related conditions from the A and B rows of the PACT suite, appeared to be in the crystallization zone of sfCherry and yielded long clustered needles or rods. The best crystals (∼200 × 20 × 10 µm) were obtained from a condition consisting of 0.1 *M* SPG (succinic acid, phosphate, glycine) buffer pH 5.0, 25%(*w*/*v*) PEG 1500. The diffraction data from these crystals contained satellite lattices, but one of the data sets was suitable for structure determination of sfCherry.

For crystallization of the sfCherry-GFP(10–11)–GFP(1–9) complex, clustered plates were observed in initial screening experiments in five conditions from rows E, F and H of the PACT suite. Subsequent optimization, including the use of glycerol as an additive to reduce nucleation, yielded diffraction-quality crystals (100 × 30 × 20 µm) from a condition consisting of 0.1 *M* bis-tris buffer pH 8.3, 20%(*w*/*v*) PEG 3350, 6%(*v*/*v*) glycerol. Fluorescence microscopy was used to verify the existence of fluorophores in the crystals. Images of crystals taken under white light and photographs of protein solutions taken with white light and under 488/520 nm and 550/610 nm excitation/emission filters are shown in Supplementary Fig. S3.

### 2.7. Data collection, molecular replacement and refinement   

Data were collected from crystals of sfCherry and the sfCherry-GFP(10–11)–GFP(1–9) complex on beamline 5.0.2 at the Advanced Light Source (ALS) and were processed with the *HKL*-2000 program (Otwinowski & Minor, 1997 ▶). The crystals of sfCherry belonged to space group *P*2_1_, with unit-cell parameters *a* = 85.105, *b* = 96.294, *c* = 105.957 Å, β = 104.56°. The data set was processed to 2.0 Å resolution with an *R*_merge_ of 9.5% and a completeness of 97.0%. Cell-content analysis gave a Matthews coefficient of 2.17 Å^3^ Da^−1^ and a solvent content of 43% with eight copies of sfCherry in the asymmetric unit. The crystals of the sfCherry-GFP(10–11)–GFP(1–9) complex belonged to space group *P*2_1_2_1_2_1_, with unit-cell parameters *a* = 74.360, *b* = 86.490, *c* = 167.941 Å. The data set for sfCherry-GFP was processed at 2.6 Å resolution with an *R*_merge_ of 6.5% and a completeness of 98.8%. The Matthews coefficient of the sfCherry-GFP(10–11)–GFP(1–9) complex crystals was 2.70 Å^3^ Da^−1^, suggesting a solvent content of 54% with two copies of the complex in the asymmetric unit.

The crystal structure of sfCherry was determined by the molecular-replacement (MR) method using the *Phaser* program (McCoy *et al.*, 2007 ▶) in the *PHENIX* suite (Adams *et al.*, 2010 ▶). The mCherry structure (PDB entry 2h5q; Shu *et al.*, 2006 ▶) was used as a search model. Model rebuilding was carried out with *AutoBuild* (Terwilliger *et al.*, 2008 ▶) and refinement with *phenix.refine* (Headd *et al.*, 2012 ▶; Afonine *et al.*, 2012 ▶). The final *R* and *R*_free_ values for sfCherry were 22.2 and 26.5%, respectively.

The crystal structure of the sfCherry-GFP(10–11)–GFP(1–9) complex was also determined with the MR method. Similar procedures and programs as those used in the sfCherry structure determination were employed but with the following differences. The sfGFP (PDB entry 2b3q; Pédelacq *et al.*, 2006 ▶) and partially refined sfCherry structures were used as search models. The sequences belonging to strands 10 and 11 of sfGFP were pruned from sfGFP and grafted between the original strands 8 and 9 of sfCherry based on the designed constructs (Fig. 4*a*). This modified sequence pair was used in model rebuilding with *AutoBuild*. Reference-structure restraints (Headd *et al.*, 2012 ▶) were used in early stages of refinement and were released at later stages. The refined structure of the sfCherry-GFP(10–11)–GFP(1–9) complex had an *R* value of 20.5% and a free *R* value of 24.9%. Detailed data-collection and refinement statistics of sfCherry and the sfCherry-GFP(10–11)–GFP(1–9) complex are listed in Table 1 ▶. The atomic coordinates and structure factors are available in the Protein Data Bank under accession codes 4kf4 for sfCherry and 4kf5 for sfCherry GFP(10–11)–GFP(1–9).

## 3. Results   

### 3.1. Strategy for modular design   

**Figure 1:**
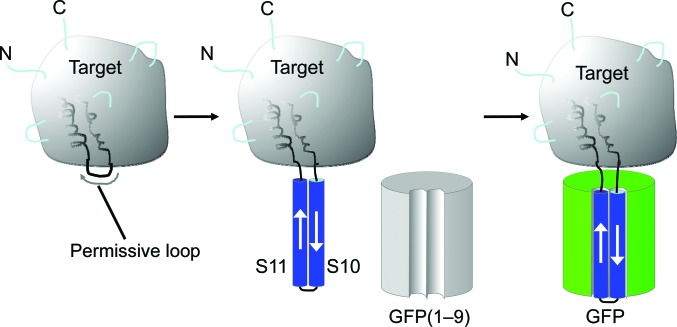
Principle of the work: insertion of GFP hairpin strands S10 and S11 into a permissive loop of a target protein, followed by reconstitution of the intact GFP by attachment of GFP(1–9) (*i.e.* the GFP molecule missing the hairpin).

The structure, stability and folding of GFP have been well studied (Örmo *et al.*, 1996 ▶; Tsien, 1998 ▶; Crameri *et al.*, 1996 ▶). Its relatively simple topology, combined with its utility as a fluorescent reporter when correctly folded (Waldo *et al.*, 1999 ▶; Pédelacq *et al.*, 2006 ▶), has made it an attractive system for reconstitution from separately expressed protein fragments (Cabantous, Terwilliger *et al.*, 2005 ▶). Following such a strategy, by fusing terminal segments of GFP to a crystallization target the resulting construct might be recombined with the remaining complementary fragment of GFP to create a new complex for crystallization. In the context of crystallization strategies, a challenge presented by typical fusion methods is the flexibility introduced at the site of connection between the two protein components; free torsion angles are present where the polypeptide backbone makes its (single) crossing from one natural protein fold to the other. The value of having the polypeptide chain cross twice instead of once between two connected proteins has been demonstrated in experiments in which T4 lysozyme was inserted into a loop of GPCR membrane proteins, giving a construct that yielded well ordered crystals (Rosenbaum *et al.*, 2007 ▶; Cherezov *et al.*, 2007 ▶). The split GFP system [GFP(1–9) + GFP(10–11)] allows a similar advantage. If strands 10 and 11, which ostensibly form a natural hairpin, can be inserted as a long extension into a surface loop of a target protein, then reconstitution with complementary GFP(1–9) should give a tight noncovalent complex with two chain crossings between natural protein folds (Fig. 1 ▶). In practice, rational choices for the points of insertion of strands 10–11 into exposed loops might be based on homology models, where available, or on bioinformatic predictions of loops (Lambert *et al.*, 2002 ▶; Dovidchenko *et al.*, 2008 ▶; Jones, 1999 ▶). Here, we chose a target for crystallization for which the structure was known, in order to test the strategy of loop insertion and crystallization in a favorable case.

### 3.2. Cherry fluorescent protein as a target protein   

In the present study, the protein chosen as a target for crystallization was superfolder Cherry (sfCherry), a version of red fluorescent protein engineered in our laboratory. sfCherry was chosen as a test protein so that the folding of the target could be monitored by red fluorescence while the GFP reconstitution could be monitored by green fluorescence. The well folding sfCherry protein was created from the fluorescent monomeric Cherry protein (mCherry; Shaner *et al.*, 2004 ▶) by directed evolution of mCherry carrying the poorly folding and aggregation-prone bullfrog red-cell H-subunit ferritin as an N-­terminal fusion, as described previously (Pédelacq *et al.*, 2006 ▶). Owing to the naturally poor folding properties of the ferritin, colonies expressing the initial ferritin-mCherry fusion at 310 K showed only faint fluorescence (Supplementary Fig. S1*a*). After three rounds of DNA shuffling, during which we selected brighter fluorescent clones expressed at 310 K, we obtained highly fluorescent ferritin-sfCherry protein fusions. *E. coli* colonies and liquid cultures of cells expressing ferritin-sfCherry fusions after three rounds of directed evolution were about 100-fold brighter than cells expressing ferritin-mCherry at 310 K (Supplementary Fig. S1*a*). Our new folding-enhanced sfCherry contains six mutations: R36H, K92T, R125L, S147T, K162N and N196D. A native polyacrylamide gel at ∼10 mg ml^−1^ protein concentration indicated that the protein is approximately 50% dimer and 50% monomer (Supplementary Fig. S1*b*).

### 3.3. Selection of a permissive insertion site in sfCherry   

**Figure 2:**
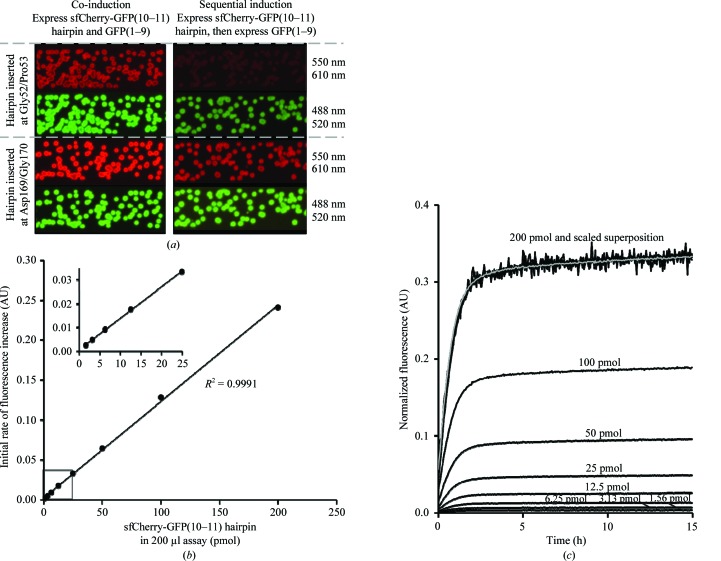
(*a*) *In vivo* protein expression (left panel) and solubility screens (right panel) for the sfCherry-GFP(10–11) hairpin inserted at Pro52/Gly53 and Asp169/Gly170. Pictures were taken of the plates after 4 h of co-induction (to monitor protein expression by GFP fluorescence) and after 2 h of induction with anhydrotetracycline (AnTet) followed by 1 h rest and 1 h induction of GFP(1–9) (to monitor soluble protein by GFP fluorescence). Fluorescence from folded sfCherry was monitored using 550 nm excitation/610 nm emission (red fluorescence) and reconstituted GFP fluorescence was monitored using 488 nm excitation/520 nm emission (green fluorescence). Pictures are shown with 0.5 s exposure times for red fluorescence and 0.25 s exposure times for green fluorescence. (*b*) *In vitro* sensitivity characterization of sfCherry-GFP(10–11) complementation with GFP(1–9). 20 µl aliquots containing 1.56–200 pmol of sfCherry-GFP(10–11) hairpin were mixed with 180 µl aliquots containing 800 pmol GFP(1–9) to start the complementation. AU, arbitrary fluorescence units. (*c*) Superimposition of scaled progress curves for complementation of 200, 100, 50, 25, 12.5, 6.25, 3.13 and 1.56 pmol samples. The curves can be superimposed well by linear scaling, indicating that the shape of the progress curves does not depend on the concentration of the tagged protein or the depletion of the pool of unbound GFP(1–9) fragment (see §[Sec sec3]3).

Our strategy of inserting GFP strands 10–11 into a target protein requires that permissive sites be identified. In order to guide the choice of sites that might be permissive for insertion into our target protein, sfCherry, we relied partly on earlier experimental data for circular permutants of superfolder GFP (sfGFP), which has 23.3% sequence identity to sfCherry and a similar structure (Pédelacq *et al.*, 2006 ▶). On this basis, the GFP(10–11) hairpin with a short linker of three residues (DAS) was inserted at two different loop sites (Gly52/Pro53 or Asp169/Gly170) of sfCherry. The three-residue linker was included to improve protein solubility as guided by our previous experiments (data not shown). These two sfCherry-GFP(10–11) hairpin constructs were screened for expression and solubility *in vivo* in *E. coli* colonies using a complementation assay with GFP(1–­9) as previously described for GFP11 and GFP(1–10) (Cabantous & Waldo, 2006 ▶). The construct with the GFP hairpin inserted at Asp169/Gly170 clearly showed brighter red and green fluorescence compared with the Gly52/Pro53 insertion (Fig. 2 ▶ *a*). We concluded that insertion of the GFP hairpin at the permissive site Asp169/Gly170 of sfCherry was the better choice for folding of the target and subsequent binding to GFP(1–9). This construct was chosen for further crystallization and structural characterization.

### 3.4. *In vitro* complementation assays of the sfCherry-GFP(10–11) hairpin with GFP(1–9) and GFP(1–10)   

To characterize sfCherry with the GFP(10–11) hairpin inserted at Asp169/Gly170 in greater detail, we complemented *in vitro* different concentrations of sfCherry in the range 1–­200 pmol (in a 20 µl aliquot) by adding a large molar excess (800 pmol in a 180 µl aliquot) of either GFP(1–9) or GFP(1–­10). As expected, the GFP(10–11) hairpin inserted into sfCherry complemented the GFP(1–9) molecule and yielded bright fluorescence (Supplementary Fig. S2*a*). Additionally, GFP(1–9) complemented the hairpin faster than did GFP(1–­10) (Supplementary Fig. S2*a*). In the latter case, strand 10 in the reconstituted GFP could come either from the hairpin [requiring the displacement of GFP strand 10 in GFP(1–­10)] or from GFP(1–10) (requiring the displacement of GFP strand 10 in the 10–11 hairpin). Potential steric hindrance from two copies of GFP strand 10 might explain the reduced kinetics with GFP(1–10), but was beyond the scope of the present work and was not explored further.

Initial complementation rates of the sfCherry-GFP(10–11) hairpin with GFP(1–9) were a linear function of the concentration of the sfCherry-GFP(10–11) hairpin (Fig. 2 ▶ *b*). The scaled complementation curves were superimposable, indicating a mechanism that is independent of the concentration of the sfCherry-GFP(10–11) hairpin (Fig. 2 ▶ *c*), as expected since GFP(1–9) was present in a large excess.

### 3.5. Crystal structure of sfCherry alone   

**Figure 3:**
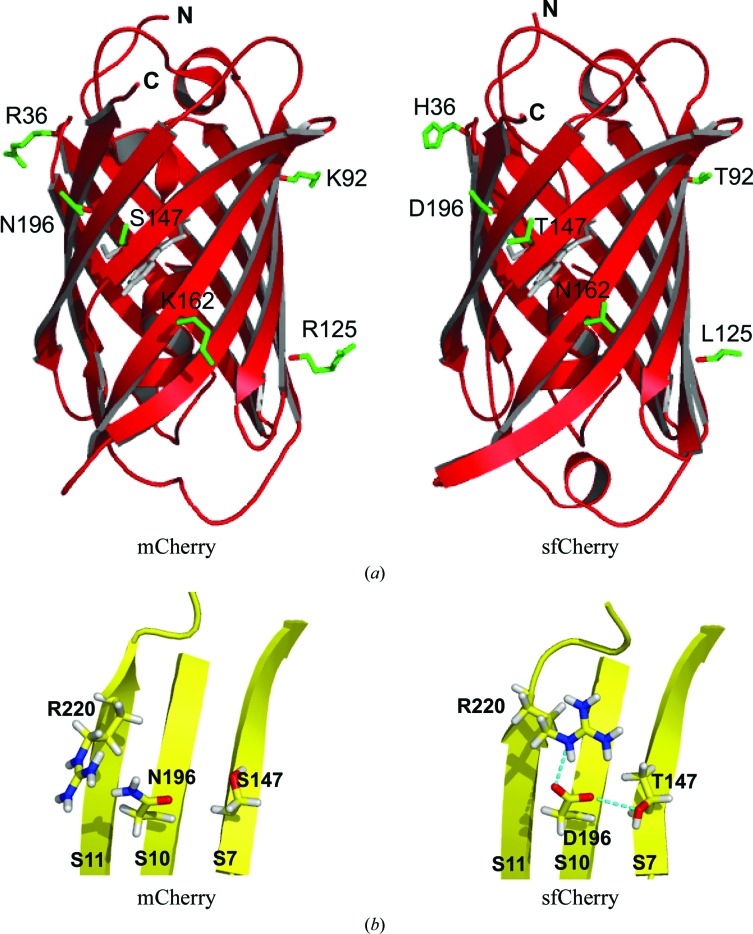
Three-dimensional structures of mCherry and sfCherry. (*a*) Structure of mCherry (left; PDB entry 2h5q; Shu *et al.*, 2006 ▶) and sfCherry (right; this work) showing the locations of sfCherry mutations and the corresponding residues in mCherry. (*b*) Region of mCherry and sfCherry close to residues 147 and 196 showing the hydrogen bonds formed between Asp196 and Thr147, and between Asp196 and Arg220 in the dimeric sfCherry structure (right) and the lack of corresponding hydrogen bonds between Asn196 and Ser147 or Arg220 in the monomeric mCherry structure (left). The images were created with *PyMOL* (DeLano, 2002 ▶)

To allow subsequent comparisons, the crystal structure of sfCherry (without a loop insertion) was determined at 2 Å resolution from the protein expressed in *E. coli* (Table 1 ▶). The C^α^ superposition of sfCherry and mCherry (PDB entry 2h5q; Shu *et al.*, 2006 ▶) has a root-mean-square deviation (r.m.s.d.) of only 0.17 Å for residues 6–223 (Fig. 3 ▶ *a*). The chromophore is formed from residues Met66-Tyr67-Gly68 and is buried in the middle of the central helix. Unlike mCherry, sfCherry crystallized as a symmetric dimer. The dimer interface includes the hydrophobic residues Val96, Val104 and Leu125 and the hydrophilic residues Asn23, Glu94, Thr106, Thr108, Thr127 and Asn128. Similar to the *AB* dimer interface found in the Dsred tetramer (Yarbrough *et al.*, 2001 ▶), the sequence Val104, Thr106, Thr108 is central to the dimer interface in the sfCherry structure, in which Thr106*A* forms a hydrogen bond to its counterpart Thr106*B*. A sequence alignment of sfCherry, mCherry and Dsred (Supplementary Fig. S2*b*) suggests that the R125L mutation in sfCherry is likely to contribute to the observed dimerization. In both the Dsred tetramer (PDB entry 1g7k) and the sfCherry structures (this work), either Ile125 (Dsred) or Leu125 (sfCherry) may stabilize the dimer through hydrophobic interactions. In the mCherry structure (PDB entry 2h5q), the bulky charged side chain Arg125 is likely to prevent dimerization by charge repulsion. In the sfCherry structure, the side chains of Asp196 form hydrogen bonds to Arg220 *via* O^δ2^ and to Thr147 *via* O^δ1^, while the corresponding interactions between Asn196 and Arg220/Ser147 are not present in the mCherry structure (Fig. 3 ▶ *b*). This change in the hydrogen-bonding network, together with the R125L mutation, may explain in part why sfCherry is more stable and more tolerant to folding interference compared with mCherry when fused to a poorly folding and aggregation-prone protein such as H-subunit ferritin (Supplementary Fig. S1*a*).

### 3.6. Structure of sfCherry with GFP strands 10–11 inserted at Asp169/Gly170 in complex with GFP(1–9)   

**Figure 4:**
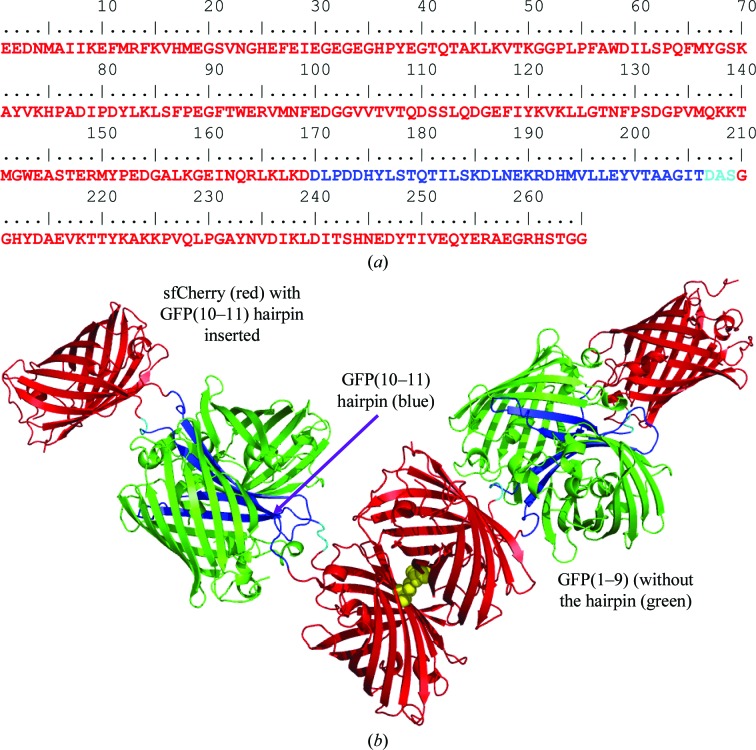
Three-dimensional structure of the sfCherry-GFP(10–11) hairpin complexed with GFP(1–9). (*a*) The amino-acid sequence of the sfCherry-GFP(10–11) hairpin is colored red for the sfCherry component, blue for the GFP(10–11) hairpin and cyan for the three-residue linker. (*b*) Structure of the sfCherry-GFP(10–11)–GFP(1–9) complex with the same color scheme used as in the amino-acid sequence. sfCherry forms dimers in the crystal through an interface involving the side chains of Thr106 (shown as spheres).

**Figure 5:**
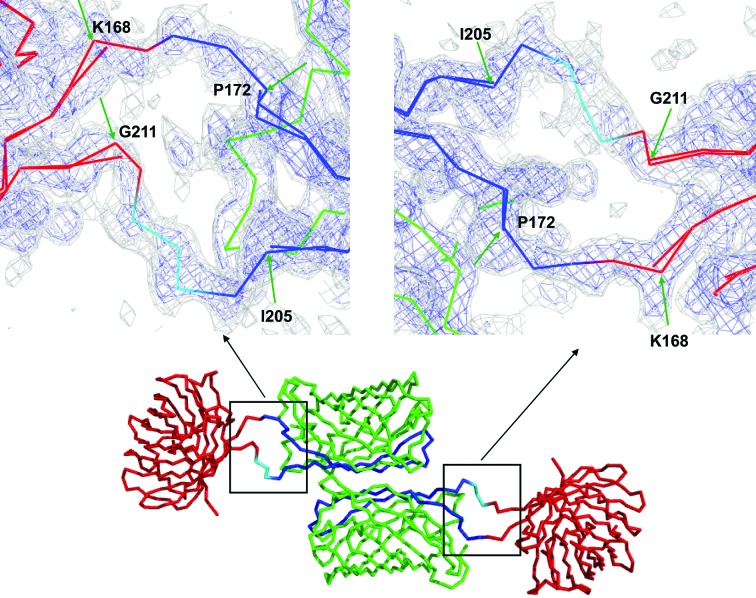
A 2*mF*_o_ − *DF*_c_ σ_A_-weighted electron-density map (Winn *et al.*, 2011 ▶) was calculated using a model that was constructed before any connections between sfCherry and the GFP(10–11) hairpin had been built. The connections between the green arrows (not included in the phasing model) are between residues 168 and 172 and residues 205 and 211. This unbiased map (contoured at 0.5σ in gray and at 1σ in blue) shows clear connections between sfCherry and the GFP(10–11) hairpin that was inserted into sfCherry at an exposed loop.

**Figure 6:**
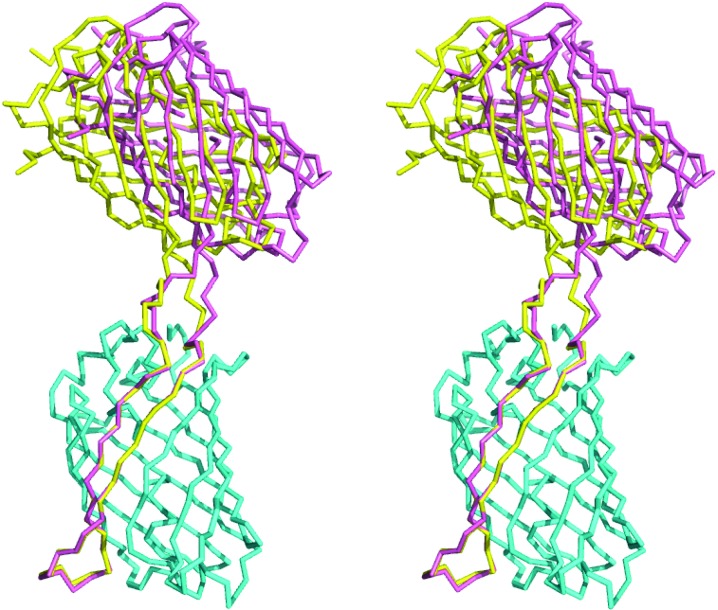
Stereoview showing an overlap of the two instances of the complex in the asymmetric unit. When the two GFP components (cyan, bottom) are superimposed, the sfCherry components in the two copies of the complex (yellow and pink) are observed to differ by only a small rotation. The images were created with *PyMOL* (DeLano, 2002 ▶).

The structure of sfCherry-GFP(10–11) in complex with GFP(1–9) was determined at 2.6 Å resolution, with final *R* and *R*_free_ values of 0.205 and 0.247, respectively (Table 1 ▶). No major elements of disorder, conformational heterogeneity or anisotropy were observed. The structure of the complex (Fig. 4 ▶ *b*) shows sfCherry to be clearly linked to the GFP(10–11) hairpin and that the GFP(10–11) hairpin complements GFP(1–9) to form an intact GFP molecule. The crystal asymmetric unit contains two copies of the complex. With two complexes in the asymmetric unit, and two linking chain segments between the two protein components in each case, there are four linking polypeptide segments. All of these segments are well ordered and clearly visible in the final electron-density map (Fig. 5 ▶). Furthermore, the relative orientation of the GFP and sfCherry components in the complex is very similar in the two instances visualized in the asymmetric unit. When the GFP components of the two independent complexes are spatially overlapped, the sfCherry components differ in the two cases by a rotation of only 9° (Fig. 6 ▶).

The GFP domains form a dimer in the crystal with local twofold symmetry (Fig. 4 ▶ *b* and Supplementary Fig. S4*a*). The GFP dimer interface is mediated through β-strand 10 (inserted in sfCherry) *via* the sequence Gln180, Ile182 and Leu183 and the loop Phe145, Asn146 and Ser147 connecting strand 6 and strand 7 of GFP(1–9). Residue Gln180 of GFP β-strand 10 is hydrogen-bonded to the backbone of its counterpart Leu183 *via* the N^∊^ and O^∊^ atoms. Position Ile182 in the sfCherry-GFP(10–11) hairpin construct corresponds to Ala206 in the folding reporter GFP and to Val206 in sfGFP (Pédelacq *et al.*, 2006 ▶). The dimer interface found in the crystal structure of the folding reporter GFP (PDB entry 2b3q) was also mediated through Gln204, Ala206 and Leu207 of strand 10 and Tyr145, Asn146 and Ser147 of the loop connecting strand 6 and strand 7 (Pédelacq *et al.*, 2006 ▶), similar to the interface found in our sfCherry-GFP(10–11)–GFP(1–9) complex structure. The sfCherry domains are arranged in the crystal as a dimer that is essentially identical to the dimer formed when crystallized by itself (Fig. 4 ▶ *b* and Supplementary Fig. S4*b*). The crystal structure exhibits strong packing interactions in all three dimensions owing to the dimerization of the reconstituted GFP, the linkage between GFP and sfCherry (creating linkages in the *xy* plane) and the dimerization of sfCherry (creating linkages in the *z* direction).

## 4. Discussion   

The purpose of these experiments was to develop a modular framework for using split GFP as a crystallization partner. Here, we present a proof-of-principle experiment in which we used GFP reconstitution to monitor the success of GFP hairpin insertion into sfCherry, a red fluorescent protein, and then characterized the atomic structure of the sfCherry-GFP(10–11)–GFP(1–9) protein complex by X-ray diffraction. We note that the GFP(10–11) hairpin described here was originally optimized as a protein-interaction detector with each β-strand separately attached to an interacting protein (Cabantous *et al.*, 2013 ▶). Part of this optimization involved eliminating any aggregation and self-assembly between the β-strands. This could potentially destabilize the GFP(10–11) hairpin prior to complementation by GFP(1–9), affecting the stability of target proteins. Despite these caveats, we found a site for insertion of the GFP(10–11) hairpin sequence that did not substantially disrupt the folding of the well folded sfCherry. However, the insertion of the GFP(10–11) hairpin might affect the stability of less stable target proteins. The choice of insertion site might therefore be important in more general applications. For choosing the permissive sites of sfCherry in this study, we relied partly on homology models (below) and partly on our previous experimental data for circular permutants of sfGFP, as indicated in §[Sec sec3.3]3.3. A homology model obtained for the sfCherry sequence using *SWISS-MODEL* (Arnold *et al.*, 2006 ▶; Guex & Peitsch, 1997 ▶; Schwede *et al.*, 2003 ▶) has an r.m.s.d. of 0.2 Å for the C^α^ atoms of residues 6–222 compared with the actual structure that we obtained for sfCherry in this study. The GFP(10–11) hairpin sequence could have been inserted into any of several loop sites of sfCherry based on this homology model; *in vivo* experiments (§[Sec sec2.2]2.2) could have been used to screen for the most permissive site. The GFP(10–11) hairpin sequence reported in this paper is likely to be suitable for insertion into various other target proteins. We are currently engineering the GFP(10–11) hairpin sequence specifically as an insertion in order to minimize the effects that it might have on the stability of target proteins.

While some structures have previously been obtained for proteins fused terminally to full-length GFP, the use of the GFP hairpin insertion instead as a fusion partner has potential benefits for crystallization. The hairpin is small and may be less perturbing of protein folding than a fusion of intact GFP. Further, the hairpin is topologically well suited for insertion into the loops and turns of a target protein. Finally, instead of the single-chain crossing afforded by terminal GFP fusions, the hairpin provides two chain crossings between the target and the reconstituted GFP. We expect this to be an important feature, as it would be expected to reduce the flexibility between the connected components. This expectation was confirmed by the crystal structure of our complex. We observed that the chain-crossing segments were well ordered. Perhaps more compellingly, the two instances of the complex seen in the asymmetric unit of the crystal suggest that the two connected components, GFP and the sfCherry target protein, sample a rather limited range of relative orientations. The relative orientation of the two components differs by 9° when the two complexes are compared. This appears essentially as a minor hinge motion through the two points of connection; twisting and rotation about the other orthogonal direction is evidently limited by the double connection. The connection therefore appears to be relatively rigid.

The ease with which the current version of GFP strands 10–­11 could be inserted into a test protein and then readily crystallized as a complex with GFP(1–9) suggests that the approach may be widely applicable, especially after further optimization of the GFP(10–11) hairpin. The case presented here held the advantage that the target protein had already been structurally characterized, so that the surface loops for insertion could be defined easily. For more realistic applications, homology modeling could be valuable in selecting prospective insertion sites. In the most challenging cases, such as where the target protein has no homologs of known structure, a library of constructs with the hairpin randomly inserted could be created and the *in vivo* solubility assay with GFP(1–9) (described in this study) could be used to screen for permissive sites.

The natural modularity of our split system for crystallization opens the possibility of engineering and testing many variants of GFP(1–9) that might be expected to have distinct crystallization behaviors. In this way, a single target protein construct bearing a GFP(10–11) insertion could be combined with any number of different variants of the GFP(1–9) carrier, leading to greatly expanded chances of crystallization. This strategy would circumvent the labor associated with exhaustively re-engineering a protein being targeted for crystallization, since the purified target protein bearing the GFP hairpin could be complemented with different pre-purified GFP(1–9) mutants without further genetic manipulation, protein expression and purification. The strategy shown here could be applied to detergent-solubilized membrane proteins, inserting the GFP(10–11) hairpin into exposed cytoplasmic loops, as well as soluble proteins. Another benefit of this system is that GFP can potentially be used as the search model in molecular replacement, making it possible to obtain diffraction phases and electron-density maps even for a target protein with an unknown fold.

Many of the techniques that have been used to vary the crystallization behavior of proteins could be employed to modify the GFP(1–9) carrier. In particular, synthetically symmetrized versions of GFP(1–9) should lead to highly distinct constructs, with each providing essentially independent opportunities for forming lattice contacts during crystallization. The creation of unique GFP(1–9) modules supporting the formation of new lattices and the development of methods to attach them to target proteins *via* engineered versions of a GFP(10–11) hairpin are ongoing projects in our laboratories.

## Supplementary Material

PDB reference: sfCherry, 4kf4

PDB reference: sfCherry-GFP(10–11)–GFP(1–9) complex, 4kf5

Supplementary figures and table.. DOI: 10.1107/S0907444913024608/dz5297sup1.pdf

## Figures and Tables

**Table 1:** Statistics of data collection and refinement for sfCherry (PDB entry 4kf4) and the sfCherry GFP(1011)GFP(19) complex (PDB entry 4kf5) Values in parentheses are for the highest resolution shell.

	sfCherry	sfCherry GFP(1011)GFP(19) complex
Data collection		
Wavelength ()	1.0	1.0
Resolution ()	50.002.00 (2.032.00)	50.002.60 (2.642.60)
No. of observations	247180	142199
No. of unique reflections	109059 (5459)	63036 (2956)
Completeness (%)	97.0 (98.1)	98.8 (94.0)
*R*_merge_ † (%)	9.5 (54.6)	6.5 (47.7)
*I*/(*I*)	10.5 (1.6)	18.8 (2.2)
Multiplicity	2.3 (2.2)	4.2 (4.2)
Refinement		
Resolution ()	50.02.00 (2.052.00)	50.02.60 (2.672.60)
*R*_cryst_ ‡ (%)	22.24 (27.74)	20.54 (32.81)
*R*_free_ ‡ (%)	26.48 (32.42)	24.89 (38.00)
R.m.s.d., bonds ()	0.008	0.005
R.m.s.d., angles ()	1.059	0.895
Average *B* value, protein (^2^)	27.9	83.2§
Average *B* value, water (^2^)	29.2	59.4
Ramachandran plot (%)		
Most favored	98.1	96.6
Allowed	1.9	3.4
Outliers	0	0

† *R*_merge_ = 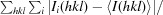 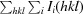 100, where *I*_*i*_(*hkl*) is the *i*th measurement of reflection *hkl* and *I*(*hkl*) is the average value of the reflection intensity.

‡ *R*_cryst_/*R*_free_ = 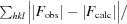 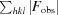 100.

§ Average *B* values by protein chain (^2^): *A*, 96.9; *B*, 98.9; *C*, 71.6; *D*, 73.2.

## Footnotes

1 Supplementary material has been deposited in the IUCr electronic archive (Reference: DZ5297).

## References

[bb1] Adams, P. D. *et al.* (2010). *Acta Cryst.* D**66**, 213–221.

[bb2] Afonine, P. V., Grosse-Kunstleve, R. W., Echols, N., Headd, J. J., Moriarty, N. W., Mustyakimov, M., Terwilliger, T. C., Urzhumtsev, A., Zwart, P. H. & Adams, P. D. (2012). *Acta Cryst.* D**68**, 352–367. 10.1107/S0907444912001308 PMC3322595 22505256

[bb3] Arnold, K., Bordoli, L., Kopp, J. & Schwede, T. (2006). *Bioinformatics*, **22**, 195–201. 10.1093/bioinformatics/bti770 16301204

[bb4] Banatao, D. R., Cascio, D., Crowley, C. S., Fleissner, M. R., Tienson, H. L. & Yeates, T. O. (2006). *Proc. Natl Acad. Sci. USA*, **103**, 16230–16235. 10.1073/pnas.0607674103 PMC1637565 17050682

[bb5] Berman, H., Henrick, K., Nakamura, H. & Markley, J. L. (2007). *Nucleic Acids Res.* **35**, D301–D303. 10.1093/nar/gkl971 PMC1669775 17142228

[bb10] Cabantous, S., Nguyen, H. B., Pédelacq, J.-D., Koraïchi, F., Chaudhary, A., Ganguly, K., Lockard, M. A., Favre, G., Terwilliger, T. C. & Waldo, G. S. (2013). *Sci Rep.* **3**, 2854. 10.1038/srep02854. 10.1038/srep02854 PMC3790201 24092409

[bb6] Cabantous, S., Pédelacq, J.-D., Mark, B. L., Naranjo, C., Terwilliger, T. C. & Waldo, G. S. (2005). *J. Struct. Funct. Genomics*, **6**, 113–119. 10.1007/s10969-005-5247-5 16211507

[bb7] Cabantous, S., Terwilliger, T. C. & Waldo, G. S. (2005). *Nature Biotechnol.* **23**, 102–107. 10.1038/nbt1044 15580262

[bb8] Cabantous, S. & Waldo, G. S. (2006). *Nature Methods*, **3**, 845–854. 10.1038/nmeth932 16990817

[bb9] Center, R. J., Kobe, B., Wilson, K. A., Teh, T., Howlett, G. J., Kemp, B. E. & Poumbourios, P. (1998). *Protein Sci.* **7**, 1612–1619. 10.1002/pro.5560070715 PMC2144054 9684894

[bb11] Cherezov, V., Rosenbaum, D. M., Hanson, M. A., Rasmussen, S. G. F., Thian, F. S., Kobilka, T. S., Choi, H.-J., Kuhn, P., Weis, W. I., Kobilka, B. K. & Stevens, R. C. (2007). *Science*, **318**, 1258–1265. 10.1126/science.1150577 PMC2583103 17962520

[bb13] Cooper, D. R., Boczek, T., Grelewska, K., Pinkowska, M., Sikorska, M., Zawadzki, M. & Derewenda, Z. (2007). *Acta Cryst.* D**63**, 636–645. 10.1107/S0907444907010931 17452789

[bb14] Crameri, A., Whitehorn, E. A., Tate, E. & Stemmer, W. P. (1996). *Nature Biotechnol.* **14**, 315–319. 10.1038/nbt0396-315 9630892

[bb15] DeLano, W. L. (2002). *PyMOL* http://www.pymol.org.

[bb16] Derewenda, Z. S. (2004). *Structure*, **12**, 529–535. 10.1016/j.str.2004.03.008 15062076

[bb17] Derewenda, Z. S. (2010). *Acta Cryst.* D**66**, 604–615. 10.1107/S090744491000644X PMC3089013 20445236

[bb18] Dong, A. *et al.* (2007). *Nature Methods*, **4**, 1019–1021. 10.1038/nmeth1118 PMC3366506 17982461

[bb19] Dovidchenko, N. V., Bogatyreva, N. S. & Galzitskaya, O. V. (2008). *J. Bioinform. Comput. Biol.* **6**, 1035–1047. 10.1142/s0219720008003758 18942165

[bb20] Eichinger, A., Nasreen, A., Kim, H. J. & Skerra, A. (2007). *J. Biol. Chem.* **282**, 31068–31075. 10.1074/jbc.M703552200 17699160

[bb21] Engel, C. K., Chen, L. & Privé, G. G. (2002). *Biochim. Biophys. Acta*, **1564**, 38–46. 10.1016/s0005-2736(02)00398-x 12100994

[bb22] Farinas, E. T., Bulter, T. & Arnold, F. H. (2001). *Curr. Opin. Biotechnol.* **12**, 545–551. 10.1016/s0958-1669(01)00261-0 11849936

[bb23] Forse, G. J., Ram, N., Banatao, D. R., Cascio, D., Sawaya, M. R., Klock, H. E., Lesley, S. A. & Yeates, T. O. (2011). *Protein Sci.* **20**, 168–178. 10.1002/pro.550 PMC3047073 21082721

[bb24] Guex, N. & Peitsch, M. C. (1997). *Electrophoresis*, **18**, 2714–2723. 10.1002/elps.1150181505 9504803

[bb25] Headd, J. J., Echols, N., Afonine, P. V., Grosse-Kunstleve, R. W., Chen, V. B., Moriarty, N. W., Richardson, D. C., Richardson, J. S. & Adams, P. D. (2012). *Acta Cryst.* D**68**, 381–390. 10.1107/S0907444911047834 PMC3322597 22505258

[bb26] Jones, D. T. (1999). *J. Mol. Biol.* **292**, 195–202. 10.1006/jmbi.1999.3091 10493868

[bb27] Kim, Y. *et al.* (2008). *Nature Methods*, **5**, 853–854. 10.1038/nmeth1008-853 PMC2678869 18825126

[bb28] Kovari, L. C., Momany, C. & Rossmann, M. G. (1995). *Structure*, **3**, 1291–1293. 10.1016/s0969-2126(01)00266-0 8747455

[bb29] Kuge, M., Fujii, Y., Shimizu, T., Hirose, F., Matsukage, A. & Hakoshima, T. (1997). *Protein Sci.* **6**, 1783–1786. 10.1002/pro.5560060822 PMC2143758 9260294

[bb30] Kwong, P. D., Wyatt, R., Desjardins, E., Robinson, J., Culp, J. S., Hellmig, B. D., Sweet, R. W., Sodroski, J. & Hendrickson, W. A. (1999). *J. Biol. Chem.* **274**, 4115–4123. 10.1074/jbc.274.7.4115 9933605

[bb31] Kwong, P. D., Wyatt, R., Robinson, J., Sweet, R. W., Sodroski, J. & Hendrickson, W. A. (1998). *Nature (London)*, **393**, 648–659. 10.1038/31405 PMC5629912 9641677

[bb32] Laganowsky, A., Zhao, M., Soriaga, A. B., Sawaya, M. R., Cascio, D. & Yeates, T. O. (2011). *Protein Sci.* **20**, 1876–1890. 10.1002/pro.727 PMC3267952 21898649

[bb33] Lambert, C., Léonard, N., De Bolle, X. & Depiereux, E. (2002). *Bioinformatics*, **18**, 1250–1256. 10.1093/bioinformatics/18.9.1250 12217917

[bb34] Lange, C. & Hunte, C. (2002). *Proc. Natl Acad. Sci. USA*, **99**, 2800–2805. 10.1073/pnas.052704699 PMC122428 11880631

[bb35] Lee, S.-Y., Lee, A., Chen, J. & MacKinnon, R. (2005). *Proc. Natl Acad. Sci. USA*, **102**, 15441–15446. 10.1073/pnas.0507651102 PMC1253646 16223877

[bb36] Levinson, N. M., Seeliger, M. A., Cole, P. A. & Kuriyan, J. (2008). *Cell*, **134**, 124–134. 10.1016/j.cell.2008.05.051 PMC2494536 18614016

[bb37] Lin, L., Hutzen, B., Li, P.-K., Ball, S., Zuo, M., DeAngelis, S., Foust, E., Sobo, M., Friedman, L., Bhasin, D., Cen, L., Li, C. & Lin, J. (2010). *Neoplasia*, **12**, 39–50. 10.1593/neo.91196 PMC2805882 20072652

[bb38] Longenecker, K. L., Garrard, S. M., Sheffield, P. J. & Derewenda, Z. S. (2001). *Acta Cryst.* D**57**, 679–688. 10.1107/s0907444901003122 11320308

[bb39] Lu, Y., Yeung, N., Sieracki, N. & Marshall, N. M. (2009). *Nature (London)*, **460**, 855–862. 10.1038/nature08304 PMC2770889 19675646

[bb40] McCoy, A. J., Grosse-Kunstleve, R. W., Adams, P. D., Winn, M. D., Storoni, L. C. & Read, R. J. (2007). *J. Appl. Cryst.* **40**, 658–674. 10.1107/S0021889807021206 PMC2483472 19461840

[bb41] Monné, M., Han, L., Schwend, T., Burendahl, S. & Jovine, L. (2008). *Nature (London)*, **456**, 653–657. 10.1038/nature07599 19052627

[bb42] Monroe, N., Sennhauser, G., Seeger, M. A., Briand, C. & Grütter, M. G. (2011). *J. Struct. Biol.* **174**, 269–281. 10.1016/j.jsb.2011.01.014 21296164

[bb43] Moon, A. F., Mueller, G. A., Zhong, X. & Pedersen, L. C. (2010). *Protein Sci.* **19**, 901–913. 10.1002/pro.368 PMC2868234 20196072

[bb44] Nasreen, A., Vogt, M., Kim, H. J., Eichinger, A. & Skerra, A. (2006). *Protein Sci.* **15**, 190–199. 10.1110/ps.051775606 PMC2242363 16322568

[bb73] Ormö, M., Cubitt, A. B., Kallio, K., Gross, L. A., Tsien, R. Y. & Remington, S. J. (1996). *Science*, **273**, 1392–1395. 10.1126/science.273.5280.1392 8703075

[bb45] Ostermeier, C., Iwata, S., Ludwig, B. & Michel, H. (1995). *Nature Struct. Biol.* **2**, 842–846. 10.1038/nsb1095-842 7552705

[bb46] Otwinowski, Z. & Minor, W. (1997). *Methods Enzymol.* **276**, 307–326. 10.1016/S0076-6879(97)76066-X 27754618

[bb47] Pantazatos, D., Kim, J. S., Klock, H. E., Stevens, R. C., Wilson, I. A., Lesley, S. A. & Woods, V. L. Jr (2004). *Proc. Natl Acad. Sci. USA*, **101**, 751–756. 10.1073/pnas.0307204101 PMC321753 14715906

[bb48] Pédelacq, J.-D., Cabantous, S., Tran, T., Terwilliger, T. C. & Waldo, G. S. (2006). *Nature Biotechnol.* **24**, 79–88. 10.1038/nbt1172 16369541

[bb49] Pédelacq, J.-D., Piltch, E., Liong, E. C., Berendzen, J., Kim, C.-Y., Rho, B.-S., Park, M. S., Terwilliger, T. C. & Waldo, G. S. (2002). *Nature Biotechnol.* **20**, 927–932. 10.1038/nbt732 12205510

[bb50] Pornillos, O., Ganser-Pornillos, B. K., Kelly, B. N., Hua, Y., Whitby, F. G., Stout, C. D., Sundquist, W. I., Hill, C. P. & Yeager, M. (2009). *Cell*, **137**, 1282–1292. 10.1016/j.cell.2009.04.063 PMC2840706 19523676

[bb51] Rosenbaum, D. M., Cherezov, V., Hanson, M. A., Rasmussen, S. G. F., Thian, F. S., Kobilka, T. S., Choi, H.-J., Yao, X.-J., Weis, W. I., Stevens, R. C. & Kobilka, B. K. (2007). *Science*, **318**, 1266–1273. 10.1126/science.1150609 17962519

[bb52] Rypniewski, W. R., Holden, H. M. & Rayment, I. (1993). *Biochemistry*, **32**, 9851–9858. 10.1021/bi00088a041 8373783

[bb53] Schwede, T., Kopp, J., Guex, N. & Peitsch, M. C. (2003). *Nucleic Acids Res.* **31**, 3381–3385. 10.1093/nar/gkg520 PMC168927 12824332

[bb54] Shaner, N. C., Campbell, R. E., Steinbach, P. A., Giepmans, B. N., Palmer, A. E. & Tsien, R. Y. (2004). *Nature Biotechnol.* **22**, 1567–1572. 10.1038/nbt1037 15558047

[bb55] Shu, X., Shaner, N. C., Yarbrough, C. A., Tsien, R. Y. & Remington, S. J. (2006). *Biochemistry*, **45**, 9639–9647. 10.1021/bi060773l 16893165

[bb56] Smyth, D. R., Mrozkiewicz, M. K., McGrath, W. J., Listwan, P. & Kobe, B. (2003). *Protein Sci.* **12**, 1313–1322. 10.1110/ps.0243403 PMC2323919 12824478

[bb57] Stemmer, W. P. (1994). *Proc. Natl Acad. Sci. USA*, **91**, 10747–10751. 10.1073/pnas.91.22.10747 PMC45099 7938023

[bb58] Suzuki, N., Hiraki, M., Yamada, Y., Matsugaki, N., Igarashi, N., Kato, R., Dikic, I., Drew, D., Iwata, S., Wakatsuki, S. & Kawasaki, M. (2010). *Acta Cryst.* D**66**, 1059–1066. 10.1107/S0907444910032944 20944239

[bb59] Terwilliger, T. C., Grosse-Kunstleve, R. W., Afonine, P. V., Moriarty, N. W., Zwart, P. H., Hung, L.-W., Read, R. J. & Adams, P. D. (2008). *Acta Cryst.* D**64**, 61–69. 10.1107/S090744490705024X PMC2394820 18094468

[bb60] Terwilliger, T. C., Stuart, D. & Yokoyama, S. (2009). *Annu. Rev. Biophys.* **38**, 371–383. 10.1146/annurev.biophys.050708.133740 PMC2847842 19416074

[bb61] Thornton, J. M. & Sibanda, B. L. (1983). *J. Mol. Biol.* **167**, 443–460. 10.1016/s0022-2836(83)80344-1 6864804

[bb62] Tsien, R. Y. (1998). *Annu. Rev. Biochem.* **67**, 509–544. 10.1146/annurev.biochem.67.1.509 9759496

[bb63] Ullah, H., Scappini, E. L., Moon, A. F., Williams, L. V., Armstrong, D. L. & Pedersen, L. C. (2008). *Protein Sci.* **17**, 1771–1780. 10.1110/ps.035121.108 PMC2548356 18715992

[bb64] Waldo, G. S. (2003). *Curr. Opin. Chem. Biol.* **7**, 33–38. 10.1016/s1367-5931(02)00017-0 12547424

[bb65] Waldo, G. S., Standish, B. M., Berendzen, J. & Terwilliger, T. C. (1999). *Nature Biotechnol.* **17**, 691–695. 10.1038/10904 10404163

[bb66] Walter, T. S., Meier, C., Assenberg, R., Au, K. F., Ren, J., Verma, A., Nettleship, J. E., Owens, R. J., Stuart, D. I. & Grimes, J. M. (2006). *Structure*, **14**, 1617–1622. 10.1016/j.str.2006.09.005 PMC7126202 17098187

[bb67] Wiltzius, J. J., Sievers, S. A., Sawaya, M. R. & Eisenberg, D. (2009). *Protein Sci.* **18**, 1521–1530. 10.1002/pro.145 PMC2775219 19475663

[bb12] Winn, M. D. *et al.* (2011). *Acta Cryst.* D**67**, 235–242.

[bb68] Yamada, H. *et al.* (2007). *Protein Sci.* **16**, 1389–1397. 10.1110/ps.072851407 PMC2206683 17586772

[bb69] Yanez, M. E., Korotkov, K. V., Abendroth, J. & Hol, W. G. J. (2008). *J. Mol. Biol.* **375**, 471–486. 10.1016/j.jmb.2007.10.035 PMC2219201 18022192

[bb70] Yarbrough, D., Wachter, R. M., Kallio, K., Matz, M. V. & Remington, S. J. (2001). *Proc. Natl Acad. Sci. USA*, **98**, 462–467. 10.1073/pnas.98.2.462 PMC14609 11209050

[bb71] Yeung, N., Lin, Y.-W., Gao, Y.-G., Zhao, X., Russell, B. S., Lei, L., Miner, K. D., Robinson, H. & Lu, Y. (2009). *Nature (London)*, **462**, 1079–1082. 10.1038/nature08620 PMC4297211 19940850

[bb72] Yip, C. K., Kimbrough, T. G., Felise, H. B., Vuckovic, M., Thomas, N. A., Pfuetzner, R. A., Frey, E. A., Finlay, B. B., Miller, S. I. & Strynadka, N. C. J. (2005). *Nature (London)*, **435**, 702–707. 10.1038/nature03554 15931226

