# Supplementary material for: Split green fluorescent protein as a modular binding partner for protein crystallization
Source: Acta Crystallogr D Biol Crystallogr. 2013 Nov 19;69(Pt 12):2513–23. doi: 10.1107/S0907444913024608 (PMC3852656; doi:10.1107/S0907444913024608)
Supplement: Supplementary file 1 [file d-69-02513-sup1.pdf]

SUPPLEMENTARY MATERIAL

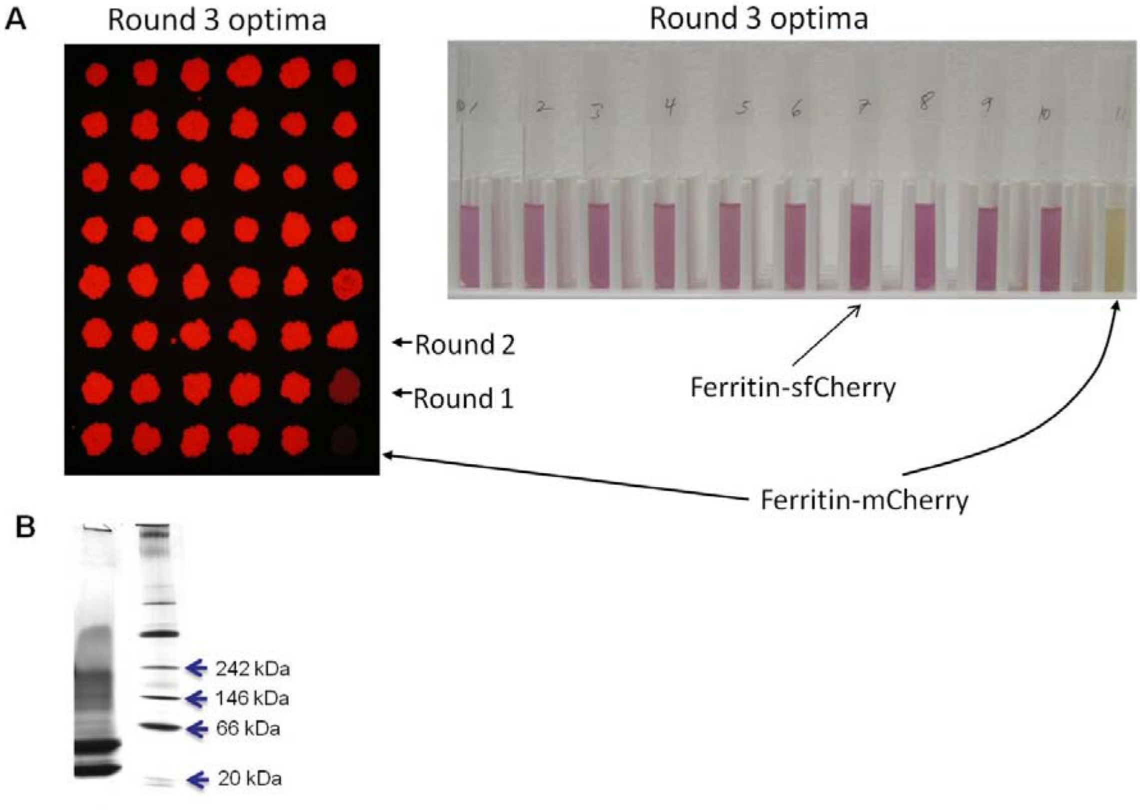

Supplementary Figure S1. (A) Fluorescence images of *E. coli* cell colonies (left) and liquid cultures (right) expressing the best ferritin-Cherry fusion constructs obtained from third round of directed evolution compared to the starting ferritin-mCherry fusion construct. (B) Native gel showing sfCherry expressed alone runs as 50% monomer: 50% dimer at concentration of ~ 10 mg/ml

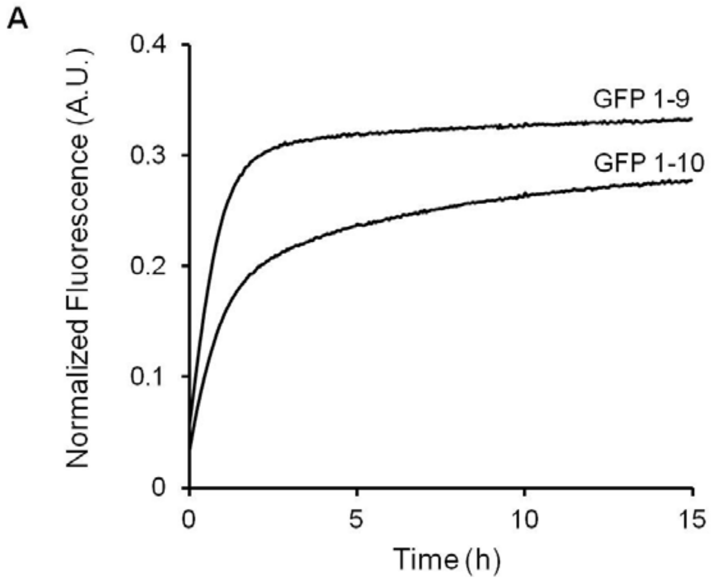

B

```
sfCherry   ~~~~~EEDNMAIIKEFMRFKVHMEGSVNGHEFEIEGEGEGHPYEGTQTAKLKVTKGGPLP    55
mCherry    MVSKGEEDNMAIIKEFMRFKVHMEGSVNGHEFEIEGEGEGRPYEGTQTAKLKVTKGGPLP    55
DsRed      MRSSKN~~~~~VIKEFMRFKVRMEGTVNGHEFEIEGEGEGRPYEGHNTVKLKVTKGGPLP    55

sfCherry   FAWDILSPQFMYGSKAYVKHPADIPDYLKLSFPEGFTWERVMNFEDGGVVTVTQDSSLQD   115
mCherry    FAWDILSPQFMYGSKAYVKHPADIPDYLKLSFPEGFKWERVMNFEDGGVVTVTQDSSLQD   115
DsRed      FAWDILSPQFQYGSKVYVKHPADIPDYKKLSFPEGFKWERVMNFEDGGVVTVTQDSSLQD   115

sfCherry   GEFIYKVKLLGTNFPSDGPVMQKKTMGWEASTERMYPEDGALKGEINQRLKLKDGGHYDA   175
mCherry    GEFIYKVKLRGTNFPSDGPVMQKKTMGWEASSERMYPEDGALKGEIKQRLKLKDGGHYDA   175
DsRed      GCFIYKVKFIGVNFPSDGPVMQKKTMGWEASTERLYPRDGVLKGEIHKALKLKDGGHYLV   175

sfCherry   EVKTTYKAKKPVQLPGAYNVDIKLDITSHNEDYTIVEQYERAEGRHSTGG~~~~~~~      225
mCherry    EVKTTYKAKKPVQLPGAYNVNIKLDITSHNEDYTIVEQYERAEGRHSTGGMDELYK*      231
DsRed      EFKSIYMAKKPVQLPGYYYVDSKLDITSHNEDYTIVEQYERTEGRHHLFL~~~~~~~      225
```

Supplementary Figure S2. (A) *In vitro* characterization of sfCherry-GFP[10-11] complementation with GFP[1-9] or GFP[1-10]. 20 µl of sfCherry-GFP[10-11] hairpin hairpin at concentration of 200 pmol per 20 µl was mixed with 180 µl aliquots containing 800 pmol of GFP[1-9] or GFP[1-10]. (B) Sequence alignment of sfCherry, mCherry and DsRed. Mutations found in sfCherry are highlighted in yellow, T106 is highlighted in cyan and L125 is highlighted in magenta.

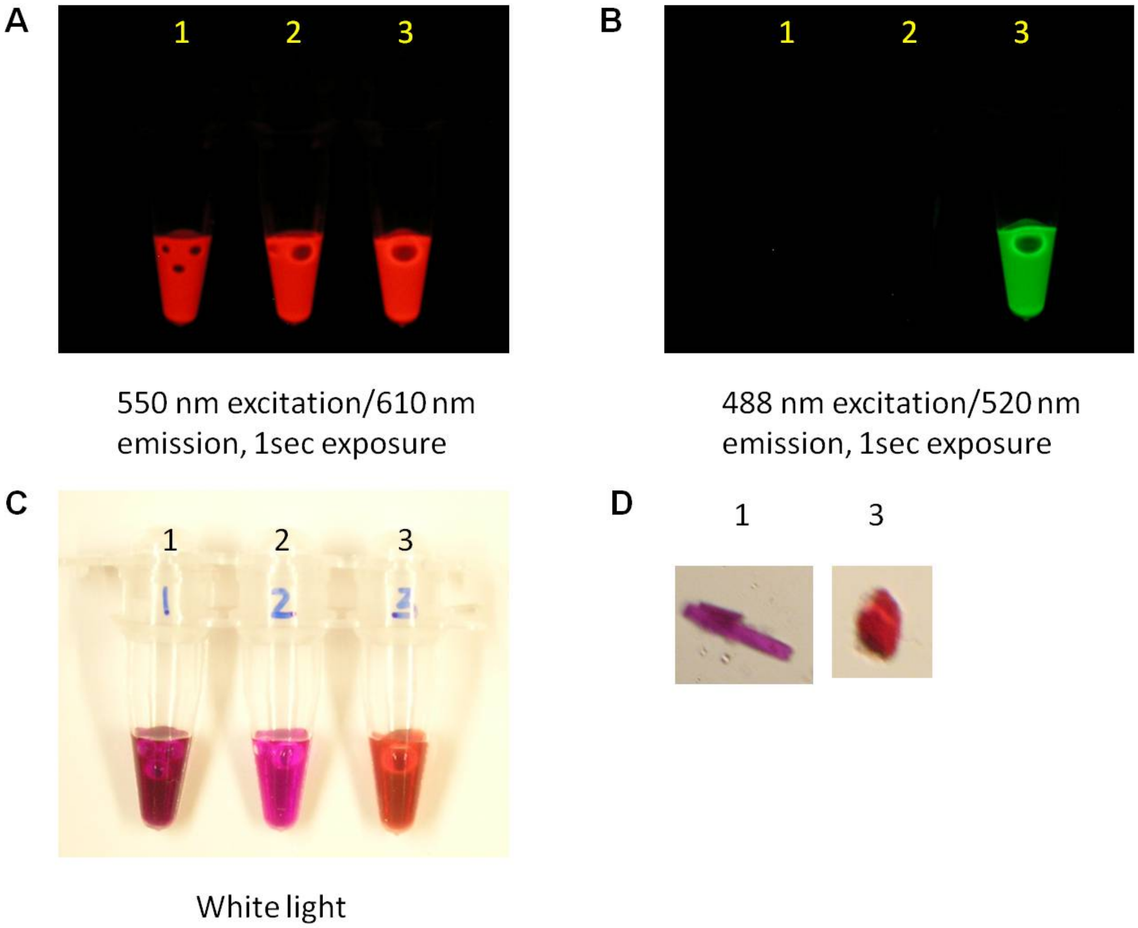

Supplementary Figure S3. Fluorescence images of sfCherry (labeled #1), sfCherry-GFP[10-11] hairpin (labeled #2), sfCherry-GFP[10-11] hairpin complemented with GFP[1-9] (labeled #3) under (A) 550 nm excitation, 610 nm emission, (B) 488 nm excitation, 520 nm emission and (C) white light. Images of sfCherry (#1) and sfCherry-GFP[10-11] hairpin complemented with GFP[1-9] (#3) crystals are shown in (D)

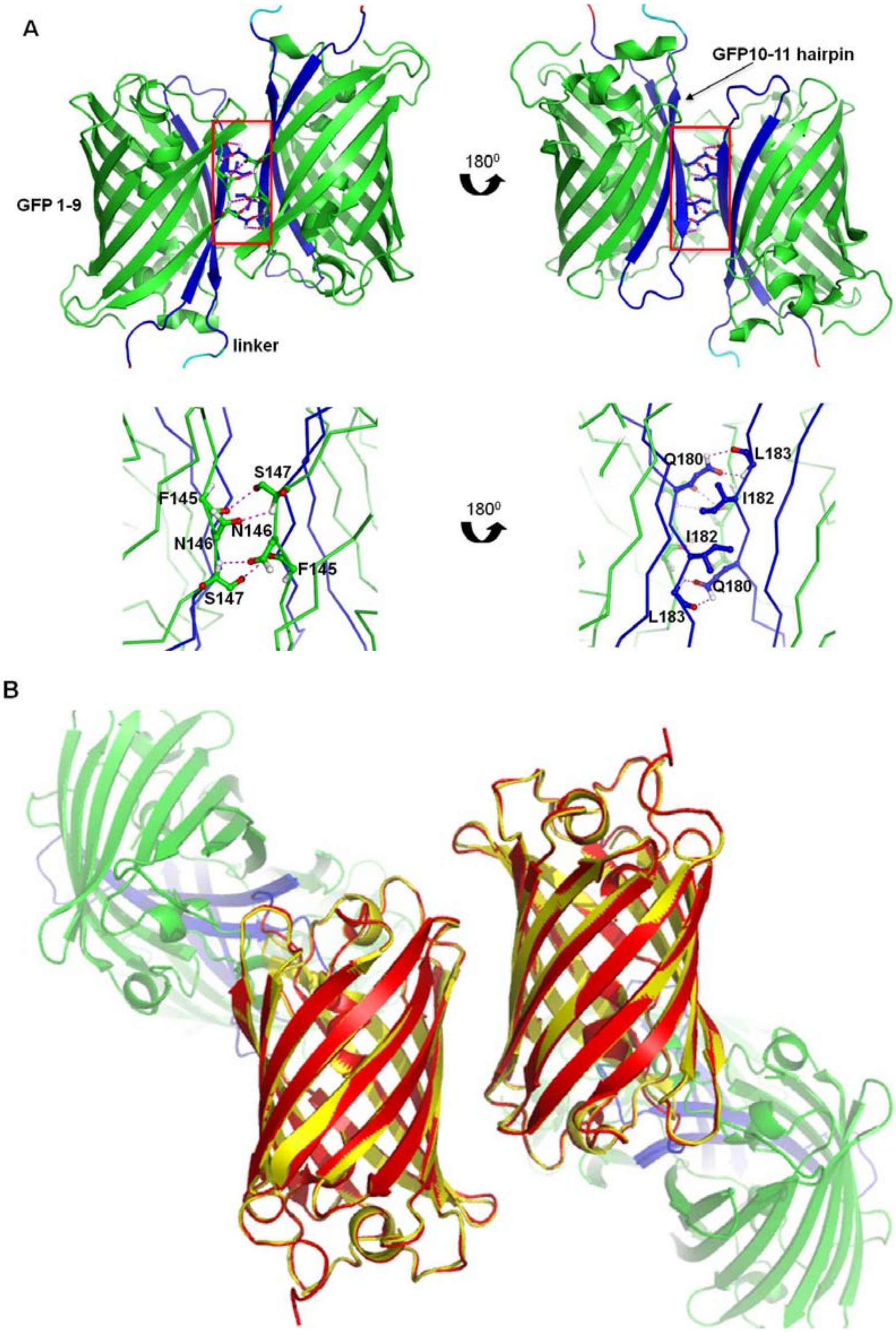

Supplementary Figure S4. (A) Dimer interface of the reconstituted GFP in sfCherry-GFP[10-11] hairpin/GFP[1-9] complex structure with involving residues labeled in balls and sticks. (B) Superimposition of sfCherry dimers in its own structure (yellow color)

with sfCherry dimers (red color) formed from lattice symmetry related sfCherry-GFP[10-11] hairpin/GFP[1-9] complex molecules

Supplementary Table S1. List of the primers used to insert GFP[10-11] hairpin into G52/P53 and D169/G170 permissive loops of sfCherry

	Primer name	Primer sequence 5' → 3'
1	G52/P53 left top	GCAAGGAATGGTGCATGCAAGGAGATGGCGCCCAACAG
2	G52/P53 left bottom 1	GACCATGTGGTCACGCTTTTCGTTGAGATCTTTCGAAAG
3	G52/P53 left bottom 2	CTTTTCGTTGAGATCTTTCGAAAGGATAGTTTGTGTCGAC
4	G52/P53 left bottom 3	TTCGAAAGGATAGTTTGTGTCGACAGGTAATGGTCGTC
5	G52/P53 left bottom 4	TGTGTCGACAGGTAATGGTCGTCTGGTAAATCTCCTCCT
6	G52/P53 left bottom 5	GTCGTCTGGTAAATCTCCTCCTTTAGTGACTTTCAATTTAG
7	G52/P53 right top 1	TCGAAAGATCTCAACGAAAAGCGTGACCACATGGTCCT
8	G52/P53 right top 2	CGAAAAGCGTGACCACATGGTCCTTCTTGAGTATGTAAC
9	G52/P53 right top 3	TGGTCCTTCTTGAGTATGTAACTGCTGCTGGGATTACAG
10	G52/P53 right top 4	GTAACTGCTGCTGGGATTACAGATGCATCTCCTCTTCCA
11	G52/P53 right top 5	ATTACAGATGCATCTCCTCTTCCATTCGCCTGGGATATAC
12	G52/P53 right bottom	GAGGCCTCTAGAGGTTATGCTAGTTATTGC
13	D169/G170 left top	GCAAGGAATGGTGCATGCAAGGAGATGGCGCCCAACAG
14	D169/G170 left bottom 1	GACCATGTGGTCACGCTTTTCGTTGAGATCTTTCGAAAG
15	D169/G170 left bottom 2	CTTTTCGTTGAGATCTTTCGAAAGGATAGTTTGTGTCGAC
16	D169/G170 left bottom 3	TTCGAAAGGATAGTTTGTGTCGACAGGTAATGGTCGTC
17	D169/G170 left bottom 4	TGTGTCGACAGGTAATGGTCGTCTGGTAAATCATCTTTAAG
18	D169/G170 left bottom 5	GTCGTCTGGTAAATCATCTTTAAGCTTGAGGCGTTGATTG
19	D169/G170 right top 1	TCGAAAGATCTCAACGAAAAGCGTGACCACATGGTCCT
20	D169/G170 right top 2	CGAAAAGCGTGACCACATGGTCCTTCTTGAGTATGTAAC
21	D169/G170 right top 3	TGGTCCTTCTTGAGTATGTAACTGCTGCTGGGATTACAG

22	D169/G170 right top 4	GTAACTGCTGCTGGGATTACAGATGCATCTGGTGGCCAT
23	D169/G170 right top 5	ATTACAGATGCATCTGGTGGCCATTACGATGCAGAGGTTA
24	D169/G170 right bottom	GAGGCCTCTAGAGGTTATGCTAGTTATTGC
